# Bing and Neel Syndrome

**DOI:** 10.1155/2012/845091

**Published:** 2012-08-26

**Authors:** S. Jennane, K. Doghmi, E. M. Mahtat, N. Messaoudi, B. Varet, M. Mikdame

**Affiliations:** ^1^Service d'Hématologie Clinique, Hôpital Militaire d'Instruction Mohammed V, Rabat, Morocco; ^2^Laboratoire d'Hématologie, Hôpital Militaire d'Instruction Mohammed V, Rabat, Morocco; ^3^Service d'Hématologie Clinique, Hôpital Necker-Enfants Malades, 75015 Paris, France

## Abstract

*Introduction*. We report the case of a Bing and Neel syndrome revealed by an isolated left ptosis. *Case Report*. a 57-year-old man was followed up since October 2003 for a typical Waldenström's macroglobulinemia. A first complete remission was obtained with chlorambucil. In August 2004, he relapsed. A second complete remission was obtained with RFC chemotherapy regimen (rituximab, fludarabine, and cyclophosphamide). In October 2009, the patient presented with an isolated left ptosis revealing a Bing and Neel syndrome. The diagnosis was suspected on MRI and confirmed by the detection in the CSF of a monoclonal IgM similar to the one found in the plasma. A quite good partial remission has been obtained after one course of RDHAP (rituximab, dexamethasone, cytarabine, and cisplatin) and 3 courses of RDHOx (rituximab, dexamethasone, cytarabine, and oxaliplatin), in addition to ten intrahectal chemotherapy injections. The treatment was followed by intensification and autologous stem cell transplantation. At D58, the patient died due to a septic shock. *Conclusion*. BNS is a rare and potentially treatable complication of WM. It should be considered in patients with neurologic symptoms and a history of WM.

## 1. Introduction

Neurological manifestations of Waldenström's macroglobulinemia (WM) are dominated by signs of hyperviscosity and the classic autoimmune neuropathies mediated by IgM. Direct central nervous system damage through a neoplastic lymphoplasmacytoid and plasma cells infiltration is rare: less than fifty cases have been described in the literature and are known as the Bing and Neel syndrome (BNS).

We report one case of a WM relapse, revealed by a pseudotumor form of a BNS. We review the literature on BNS and discuss its epidemiology, presentation, diagnosis, treatment, and prognosis.

## 2. Case Presentation

A 57-year-old man, heavy smoker, was treated since November 2003 for a WM. It has been clinically revealed by cervical lymphadenopathy, general signs, and dysglobulinemia purpura and biologically by an inflammatory syndrome and IgM kappa monoclonal gammopathy. The bone marrow biopsy revealed the presence of a lymphoplasmacytoid and plasma cells infiltration.

A first complete remission was obtained with chlorambucil alone. Two years later (April 2006), the patient relapsed. He got the same clinical and biological symptoms and an increased rate of IgM kappa. A second complete remission was obtained with 3 courses of rituximab and 3 courses of RFC (rituximab, fludarabine, and cyclophosphamide).

After thirty months (October 2009), the patient presented with a sudden isolated left ptosis, without general signs neither other neurological symptoms. Biologically, there was a slow and a quiet rise of the serum monoclonal IgM level but without inflammatory syndrome. MRI showed an infiltration of the left third cranial nerve, optic nerve, intraconal fat, and the presence of two T2 hyperintense left temporal lesions. Examination of the cerebrospinal fluid (CSF) showed a lymphocytic meningitis (60 cells/mm^3^), an increase of the CSF protein (2 g/l), and a normal CSF glucose. The CSF protein electrophoresis revealed the presence of a monoclonal IgM kappa similar to the plasma's one. The staging including full body scan, bone marrow aspiration, and bone marrow biopsy was normal.

Therefore, it is a matter of a relapse of a Waldenström's macroglobulinemia revealed by a pseudotumoral form of Bing and Neel syndrome.

The patient was treated with one course of R-DHAP (rituximab, dexamethasone, cytarabine, and cisplatin) which was complicated by renal failure. Thus, the cisplatin was replaced by the oxaliplatin, and the patient was given three courses of R-DHAOx (rituximab, dexamethasone, cytarabine, and oxaliplatin) and two courses of high-dose methotrexate, in addition to ten intrathecal chemotherapy injections (cytarabine, methotrexate, and methylprednisolone).

The evolution was characterized by the disappearance of ptosis and a marked regression of lesions on MRI and normalization of CSF analysis.

Considered as a very good partial response, the treatment was completed by an autologous peripheral stem cell transplantation (ASCT) conditioned with the association of cyclophosphamide, thiotepa, and busulfan. The evolution was unfavorable: the patient died fifty eight days after the autologous transplantation from septic shock secondary to prolonged febrile neutropenia.

## 3. Discussion

In 1936, eight years before the discovery of WM, Bing and Neel reported the first cases of central nervous system damage associated with hyperglobulinemia [1].

The BNS refers to cases of WN accompanied by neurological symptoms due to infiltration of the central nervous system with a neoplastic lymphoplasmacytoid and plasma cells process [2]. Publications on the BNS are rare. According to Drouet et al. [3], only 36 cases were reported in the literature [3]. Our case is the second one reported in Morocco [4].

BNS often occurs during the evolution of WN but can be its revelation mode in a third of cases [3]. BNS can take schematically two clinical forms: infiltrative form and pseudotumoral form. The infiltrative form is the most common one [3]. Its clinical presentation varies depending on the site and the infiltration extent. It can be revealed by dementia, confusion, cranial nerve deficit [5, 6], or by a conus medullaris syndrome [3].

According to our research, an impairment of the third cranial nerve has never been reported in the literature. However, there are two observations that describe impairment of other cranial nerves due to an infiltrative form of BNS. The first case describes a bilateral infiltration of the sixth cranial nerve [5] and the second describes a diffuse involvement of the orbit and the optic nerve [6].

Brain and spine MRI are useful to locate the lymphoplasmacytic infiltration and to specify the form of the BNS. It can also distinguish it from ischemic lesions and demyelinating lesions that are due to hyperviscosity [7]. In some cases, these MRI images can be mixed up with disseminated meningoencephalitis infections, like tuberculosis [3], or with a large B-cell lymphoma associated with WN [8].

The diagnosis is confirmed by the presence in the CSF of a lymphocytes clonality or a monoclonal IgM identical to the one found in the plasma [7]. Corticomeningeal biopsy is sometimes necessary. According to the literature, half of the reported cases were diagnosed after death by histological study of cerebral lesions [3].

There is still no consensus on the treatment of BNS. All the recently reported cases were treated by chemotherapy. Radiotherapy was used in localized lesions [9]. The cytotoxic agents used are the ones that are active in WN and cross the blood-brain barrier. Rituximab has been used for the first time by Drouet et al. [3], but its efficiency was not proven.

The prognosis of SBN is severe; however, some authors have reported prolonged complete remission [8, 10–12].

## 4. Conclusion

BNS is a rare and potentially treatable complication of WM. It should be considered in patients with neurologic symptoms and a history of WM.
